# The neuropathology of intimate partner violence

**DOI:** 10.1007/s00401-023-02646-1

**Published:** 2023-10-28

**Authors:** Kristen Dams-O’Connor, Alan C. Seifert, John F. Crary, Bradley N. Delman, Marc R. Del Bigio, Gabor G. Kovacs, Edward B. Lee, Amber L. Nolan, Ariel Pruyser, Enna Selmanovic, William Stewart, Emma Woodoff-Leith, Rebecca D. Folkerth

**Affiliations:** 1 Department of Rehabilitation and Human Performance, Brain Injury Research Center of Mount Sinai, Icahn School of Medicine at Mount Sinai, New York, NY USA; 2 Department of Neurology, Icahn School of Medicine at Mount Sinai, New York, NY USA; 3 Department of Diagnostic, Molecular and Interventional Radiology, Biomedical Engineering and Imaging Institute, Graduate School of Biomedical Sciences, Icahn School of Medicine at Mount Sinai, New York City, NY USA; 4 Department of Pathology, Molecular, and Cell Based Medicine, Ronald M. Loeb Center for Alzheimer’s Disease, Friedman Brain Institute, Icahn School of Medicine at Mount Sinai, New York, NY USA; 5 Nash Family Department of Neuroscience, and Artificial Intelligence & Human Health, Ronald M. Loeb Center for Alzheimer’s Disease, Friedman Brain Institute, Icahn School of Medicine at Mount Sinai, New York, NY USA; 6 Neuropathology Brain Bank & Research CoRE, Mount Sinai Hospital, New York, NY USA; 7 Department of Pathology, Rady Faculty of Health Sciences, University of Manitoba, Room 401 Brodie Centre, 727 McDermot Avenue, Winnipeg, MB Canada; 8 Diagnostic Services - Pathology, Shared Health Manitoba, Winnipeg, MB Canada; 9 Tanz Centre for Research in Neurodegenerative Disease (CRND) and Department of Laboratory Medicine and Pathobiology, Krembil Discovery Tower, University of Toronto, 60 Leonard Ave, Toronto, ON Canada; 10 Laboratory Medicine Program and Krembil Brain Institute, University Health Network, Toronto, ON Canada; 11 Translational Neuropathology Research Laboratory, Department of Pathology and Laboratory Medicine, University of Pennsylvania, Philadelphia, PA USA; 12 Department of Laboratory Medicine and Pathology, University of Washington, Seattle, WA USA; 13 Department of Neuropathology, Elizabeth University Hospital, Glasgow, G514TF Queen UK; 14 School of Psychology and Neuroscience, University of Glasgow, Glasgow, G128QQ UK; 15 Office of Chief Medical Examiner, 520 First Avenue, New York, NY 10116 USA; 16 Department of Forensic Medicine, New York University Grossman School of Medicine, New York, NY USA

**Keywords:** Cerebrovascular disease, Diffuse axonal injury, Domestic violence, Neurodegeneration, Chronic traumatic encephalopathy, Traumatic brain injury

## Abstract

Lifelong brain health consequences of traumatic brain injury (TBI) include the risk of neurodegenerative disease. Up to one-third of women experience intimate partner violence (IPV) in their lifetime, often with TBI, yet remarkably little is known about the range of autopsy neuropathologies encountered in IPV. We report a prospectively accrued case series from a single institution, the New York City Office of Chief Medical Examiner, evaluated in partnership with the Brain Injury Research Center of Mount Sinai, using a multimodal protocol comprising clinical history review, ex vivo imaging in a small subset, and comprehensive neuropathological assessment by established consensus protocols. Fourteen brains were obtained over 2 years from women with documented IPV (aged 3rd–8th decade; median, 4th) and complex histories including prior TBI in 6, nonfatal strangulation in 4, cerebrovascular, neurological, and/or psychiatric conditions in 13, and epilepsy in 7. At autopsy, all had TBI stigmata (old and/or recent). In addition, white matter regions vulnerable to diffuse axonal injury showed perivascular and parenchymal iron deposition and microgliosis in some subjects. Six cases had evidence of cerebrovascular disease (lacunes and/or chronic infarcts). Regarding neurodegenerative disease pathologies, Alzheimer disease neuropathologic change was present in a single case (8th decade), with no chronic traumatic encephalopathy neuropathologic change (CTE-NC) identified in any. Findings from this initial series then prompted similar exploration in an expanded case series of 70 archival IPV cases (aged 2nd–9th decade; median, 4th) accrued from multiple international institutions. In this secondary case series, we again found evidence of vascular and white matter pathologies. However, only limited neurodegenerative proteinopathies were encountered in the oldest subjects, none meeting consensus criteria for CTE-NC. These observations from this descriptive exploratory study reinforce a need to consider broad co-morbid and neuropathological substrates contributing to brain health outcomes in the context of IPV, some of which may be potentially modifiable.

**Supplementary Information:**

The online version contains supplementary material available at 10.1007/s00401-023-02646-1.

## Introduction

Traumatic brain injury (TBI) is recognized as one important, potentially modifiable risk factor for neurodegenerative disease, with an estimated 15–16% [13, 32, 60] of dementia in the community attributable to TBI. The relationship between TBI and lifelong brain health has attracted particular attention through recognition of the high risk of neurodegenerative disease among elite male contact sports athletes with exposure to TBI and repetitive head impact (RHI), among whom a specific pattern of neuropathology, termed chronic traumatic encephalopathy, has been found [8, 29–31, 33, 48]. Chronic traumatic encephalopathy neuropathologic change (CTE-NC) is characterized by a patchy deposition of hyperphosphorylated tau (p-tau) in neurons, at the depths of cortical sulci, typically in a perivascular distribution, with or without colocalized glial p-tau in thorn-shaped astrocytes [7].

TBI is of course not limited to the sports world. An estimated one-third of women worldwide experience physical and/or sexual intimate partner violence (IPV) during their lifetime [51], the majority of whom have sustained at least one TBI [56], though multiple TBIs are typical [63]. Nevertheless, in contrast to contact sport and wider community TBI exposures, remarkably little is known about the lifelong brain health and neuropathological sequelae of IPV-related TBI. In contrast to the growing literature describing late neuropathologies among former athletes, only isolated case reports describe outcomes in those exposed to IPV. Thus, more than 3 decades ago, features consistent with CTE-NC were described following autopsy examination of a so called “punch-drunk wife” [47] and, more recently, in a young woman with a mixed exposure history of both IPV and contact sports exposure [17].

A narrow focus on RHI and CTE-NC would fail to consider the complex patterns of brain injury exposure experienced by IPV survivors, which might include TBI (i.e., an external force to the head, neck or face that results in altered mental status) [3], RHI (i.e., cumulative exposure to repeated injuries to the head, neck or face that may not meet TBI diagnostic criteria), and nonfatal strangulation (NFS, i.e., any injury that alters cerebral blood flow secondary to manual or ligature strangulation, suffocation, or nonfatal drowning) [39]. These distinct injury etiologies, which commonly co-occur and can escalate over time among those living with IPV, beg the question of how to recognize their neuropathological signatures. For example, diffuse traumatic axonal injury secondary to rotational/torsional force is a hallmark of isolated TBI [42], and microvascular lesions are commonly observed [14, 50]. NFS-related blood pressure fluctuation can strain vessel elasticity, resulting in microvascular injury, microhemorrhage, compromised cerebral blood flow, and stroke [12, 34, 62]. Additionally, factors other than brain injury such as high rates of cardiovascular and cerebrovascular disease [11, 45], concurrent alcohol or substance use, and HIV infection [21, 43], alongside limited care-seeking and poor treatment adherence [35], may confer additional detriment to brain health.

In light of these complex factors, we sought to characterize the pathological substrate(s) of multi-etiology IPV-related brain injury with particular emphasis on neurodegenerative and vascular pathologies.

## Methods

### Overview

We performed neuropathological examinations on a prospective, discovery sample of brains of individuals with a recent and/or remote history of IPV through partnership between the New York City Office of the Chief Medical Examiner (NYC OCME) and the National Institutes of Health-funded Late Effects of TBI (LETBI) study [18]. Findings from this case series then prompted exploration in a second series of cases collated from the Province of Manitoba and multiple international archives via the CONNECT-TBI program [53]. Study procedures were approved and/or deemed not human subjects research (NHSR) by the Mount Sinai Program for the Protection of Human Subjects; cases without informed consent for research were deemed NHSR by the New York State Department of Health and Mental Hygiene Institutional Review Board and NYC OCME Legal Department.

### Initial case series

Cases were prospectively identified over a 24-month period (2020–2021) in the NYC OCME based on the following criteria: (1) females with a history of IPV documented at the time of medicolegal death investigator review (with or without reports generated separately by police), available recent/remote medical records, and/or family interview; and (2) absence of decomposition and/or postmortem intervals > 72 h (since last known to be alive). Subjects fell under the jurisdiction of the OCME as part of its public health mandate to investigate deaths, especially those not attributable to known medical illness or with evidence of violence and/or toxicologic exposure(s), from the catchment area of the Manhattan office which corresponded to a total of 948 autopsies of women aged 18 years or older over that interval. IPV-associated brain specimens were submitted prospectively to the OCME Neuropathology Service (led by RDF) for diagnosis.

For annotation of clinical history, a combination of approaches was employed. At minimum, all cases entering the OCME system were evaluated by certified Medicolegal Death Investigators, who perform scene investigations of the decedent, discussions on site with next-of-kin (NOK), documentation of medications or other substances at the scene, and procurement of medical records from healthcare facilities. Medical Examiners performed complete autopsies to document all injuries and natural disease, supported by ancillary tests necessary for determination of the cause and manner of death. LETBI staff at Mount Sinai (led by KDOC) attempted contact with known NOK of all cases for whom any NOK contacts were available for informed consent. (Of note, IPV cases resulting from homicide are especially challenging to consent, as often the perpetrators *are* the NOK.) Informants were invited to participate in a postmortem semi-structured informant interview [18, 46] to query lifetime IPV and head trauma exposure, health comorbidities, and informant characterization of cognition, motor function, mood, and functional independence, per National Institutes of Health Common Data Elements protocols for TBI-related neurodegeneration [46]. Informant interviews were completed for four cases, and medical and forensic records were available for all cases. All available records and data were reviewed (by KDOC, AP, ES, and RDF) to confirm head/brain trauma exposure characterization and medical, psychological, and other comorbidities.

All specimens were fixed in 20% formalin for 1–2 weeks, and then either cut at NYC OCME using routine examination protocols (described below) or transferred to 10% formalin. Four whole brain specimens underwent ex vivo imaging and sectioning protocols [18], as detailed previously [57]. Each imaging dataset was reviewed by a board-certified CAQ neuroradiologist (BND) with expertise in the interpretation of ex vivo MRI. Findings of potential interest were marked for histologic sampling.

In all cases, histologic sampling followed standardized protocols for evaluation of neurodegenerative disease, including CTE, with histochemical and immunohistochemical staining as previously reported [7, 40]. Briefly, for each case, a minimum of 16 anatomical regions were evaluated, consistent with consensus recommendations for evaluation of CTE-NC and Alzheimer’s disease-NC [7, 36]. Sections from these regions were then prepared for immunocytochemistry employing antibodies to p-tau (AT8, Invitrogen, catalogue # ENMN1020, concentration 1:1000), amyloid-beta (6E10, BioLegend, catalogue # 803,015, concentration 1:5000), phospho-TDP ((1D3): BioLegend, catalogue # 829,901, concentration 1:5000), and alpha-synuclein (MilliporeSigma, catalogue # MABN824MI, concentration 1:3000). Additional sections were prepared for Perls’ Prussian blue stain for hemosiderin using the standard protocols. Immunostains for amyloid precursor protein (APP; monoclonal antibody clone 22C11, Millipore Cat#MAB348) and CD68 (KP-1; monoclonal antibody, Ventana REF#790–2931) [25] were performed, and immunostain for fibrinogen was used to detect blood–brain barrier dysfunction (mouse monoclonal antibody, Abcam, Cat#AB58207) per the manufacturer’s directions.

### Secondary exploration in archival specimens

To explore the generalizability of our findings from the initial prospective case series, we identified women with IPV exposure from retrospectively available autopsy case series, from files of the Province of Manitoba and from the collaborative CONNECT-TBI (see Supplementary Tables). The same standardized methods for evaluation of neurodegenerative disease pathologies described above were employed in assessment of all CONNECT-TBI cases [7, 53]. Of the 61 Manitoba-derived cases with documented history of IPV, 29 were fixed for complete examination [7, 53] by a neuropathologist; p-tau immunostains were performed on 4–7 brain regions. The remaining 32 brains had been photographed before and after slicing fresh by a forensic pathologist; sampling for histology was limited to 1–4 samples. The neuropathologist retrospectively reviewed the history, gross photographs, H&E stained slides, as well as p-tau immunostains on the hippocampus and medial temporal ± frontal cortex. All neuropathology evaluations in both the initial and archival case series were performed by reviewers blind to patient demographic and clinical information.

### Statistical analysis

No statistical analysis plan was pre-specified for this case series. Thus, demographic characteristics, substance use history, psychiatric symptoms, medical history, injury history (remote and recent), and features of neurodegeneration are presented in a descriptive format. This exploratory study is intended to be hypothesis-generating for targeted investigations in larger samples, including appropriate prospective controls.

## Results

### Initial case series

#### Demographic and clinical information

Fourteen brains were collected prospectively from 2020 to 2021 (Table 1), ranging in age from 20s to 70s (median, 35 years; exact ages not provided to protect privacy). Eight cases were Latina, 4 were Black, and 2 were White. Nine of 14 had alcohol and/or substance use disorders; 7 had known psychiatric disorders (e.g., anxiety, depression, bipolar disorder), and 7 had epilepsy (Table 1). Four had known histories of NFS. Six had a known history of TBI; all others had documented evidence of physical IPV but insufficient detail to characterize head trauma exposure. Other known prior injuries included extremity and facial injury, and severe abrasions (one—from fleeing the abusive partner—requiring skin grafts). The five eldest women had known hypertensive, atherosclerotic, or other heart disease (three with pacemakers).

**Table 1:** Clinical histories of initial prospective IPV case series per available data

Case	Age (decade)	Ethnicity	Prior TBI	Nonfatal strangulation	Epilepsy	Mental health disorder	Other medical conditions	Cause and manner of death	Postmortem family interview
NY1	3rd	H	–	–	–	–	–	Cause: Blunt force trauma of the head Manner: Homicide	–
NY2	3rd	B	–	–	–	–	HIV/AIDS; COVID–19	Cause: COVID–19 Manner: Natural	+
NY3	3rd	W	+	–	–	SUD	Asthma; pregnancy	Cause: Acute intoxication by the combined effects of fentanyl, heroin, and cocaine Manner: Accident (substance use)	+
NY4	4th	H	–	+	–	AUD; SUD; depression; anxiety disorder; ADHD	Graves’ disease, s/p radiotherapy, with hypothyroidism	Cause: Positional asphyxia due to lodgment and suspension within garbage chute complicating acute intoxication by the combined effects of cocaine and ethanol Manner: Accident	–
NY5	4th	H	–	–	–	–	HIV/AIDS; prior meningitis; pregnancy	Cause: Multiple blunt trauma Manner: Undetermined	–
NY6	4th	H	+	+	–	AUD; SUD	Asthma	Cause: Acute fentanyl intoxication Manner: Accident (substance use)	+
NY7	4th	B	+	+	+	AUD	–	Cause: Homicidal asphyxia including compression of neck Manner: Homicide	–
NY8	4th	H	+	+	+	SUD; bipolar disorder	–	Cause: Asphyxia including neck and chest compression Manner: Homicide	–
NY9	5th	H	–	–	+	AUD; anxiety disorder	Asthma; COVID–19	Cause: Undetermined Manner: Undetermined	–
NY10	5th	B	–	–	–	SUD; bipolar disorder	HIV/AIDS; hypertension; CAD; asthma	Cause: Probable viral meningoencephalitis Manner: Natural	–
NY11	6th	W	–	–	+	SUD; anxiety disorder	HIV/AIDS; hypertension; CHD; pacemaker	Cause: Complications of acute intoxication including methadone Manner: Accident (substance use)	–
NY12	6th	H	–	–	+	–	Hypertension; Hepatitis C; DM; AVR; pacemaker	Cause: Complications of chronic and acute substance use including cocaine Manner: Accident (substance use)	–
NY13	7th	H	+	–	+	AUD; depression	DM; HLD	Cause: Chronic alcohol use Manner: Natural	–
NY14	8th	B	+	–	+	Depression	Remote temporal lobectomy for post–traumatic epilepsy; hypertension; pacemaker; “memory loss”	Cause: Hypertensive and atherosclerotic cardiovascular disease Manner: Natural	–

Data were obtained from available medical records, scene investigations, and/or next-of-kin. Data reported herein do not reflect objective postmortem findings; see Table 2 for postmortem data. See Supplement for details of findings in archival cohorts

+  assessed and present, – assessed and evidence not found, *ADHD* attention deficit-hyperactivity disorder, *AUD* alcohol use disorder, *AVR* aortic valve replacement, *B* Black, *CAD* coronary artery disease, *CHD* congenital heart disease, *DM* diabetes mellitus, *H* Hispanic/Latina, *HIV/AIDS* human immunodeficiency virus/acquired immune deficiency syndrome, *HLD* hyperlipidemia, *NY#* from New York City Office of Chief Medical Examiner, *SUD* substance use disorder, *W* White

See text for further explanation and definitions used

#### Ex vivo* neuroimaging*

**Fig. 1:**
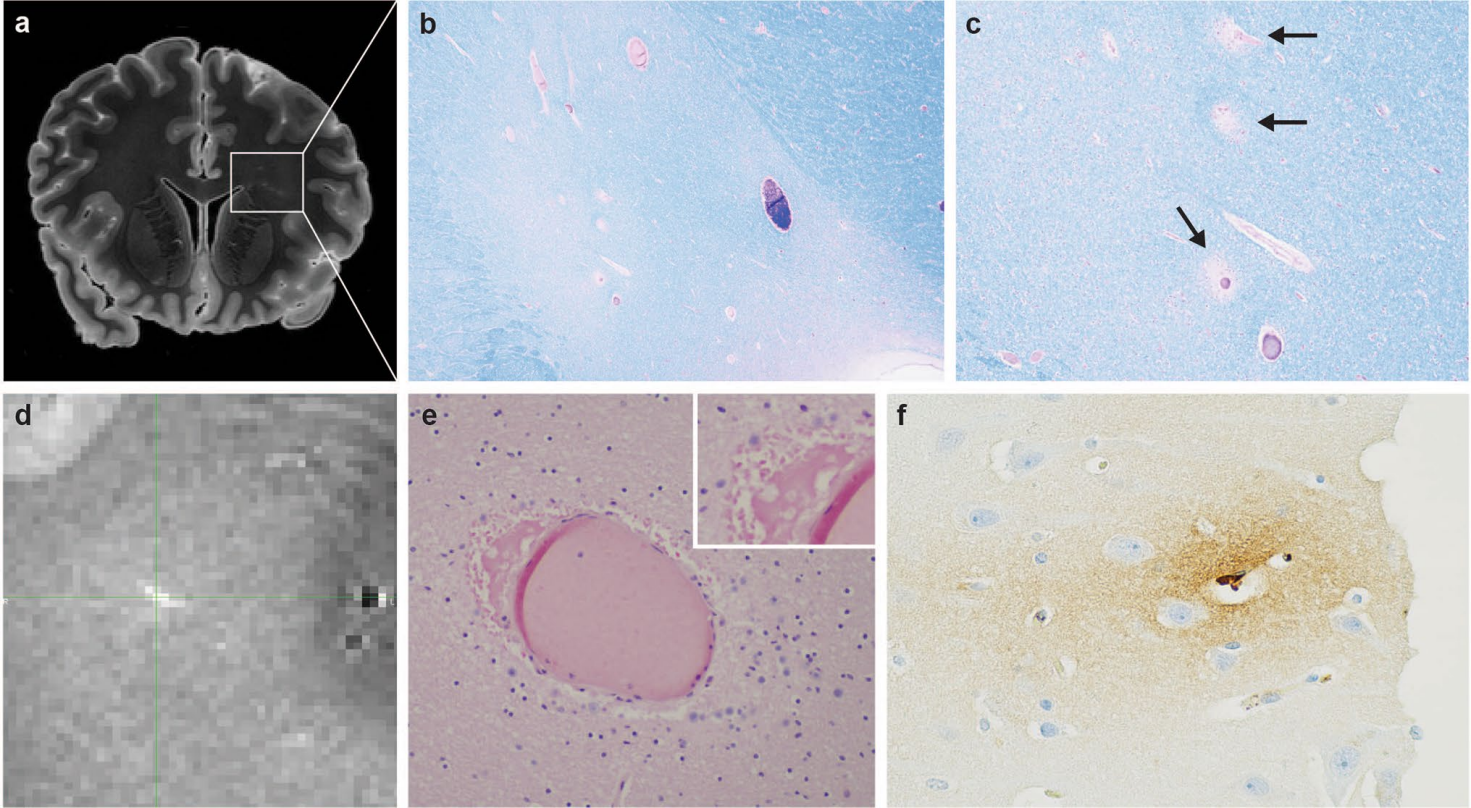
Ex vivo neuroimaging and detection of subtle vascular neuropathology in IPV. Neuroimaging of frontal lobes at level of striatum (NY6), revealing signal abnormalities in the periventricular white matter (**a**., left per neuroradiologic convention; box outlines left frontal mirror-image areas of histology in **b**., **c**.). Neurohistology of boxed area in a., demonstrating ill-defined zone of myelin pallor on Luxol-fast blue/hematoxylin-and-eosin (**b**., 20x), as well as localized perivascular rarefaction indicating loss of vascular integrity (arrows) (**c**., 40x). T1 map of different periventricular white matter region, with signal features of perivascular edema (**d**.), corresponding to perivascular accumulation of proteinaceous fluid in perivascular space (**e**., 200x); inset, detail of eosinophilic perivascular fluid, indicating vasogenic edema (400x). Immunostain for fibrinogen indicating leakage of plasma protein (brown reaction product, seen in vessel lumen) into perivascular neuropil (**f**., 200x)

Four prospective cases (all third or fourth decade in age) underwent ex vivo neuroimaging (Table 2). (The findings of this exploratory step are reported descriptively herein, with their potential import addressed in the Discussion.) Briefly, Case NY6 showed appreciable signal in the periventricular white matter, the neurohistologic substrate of which was subtle but definitive perivascular rarefaction and proteinaceous (fibrinogen-immunoreactive) fluid accumulation (Fig. 1). This acute finding, unaccompanied by inflammation, reflects the grossly observed acute TBI in this woman, who also had a history of prior TBI and NFS (see below). Case NY2 had an old left inferior temporal lobe infarct, and susceptibility changes of frontal periventricular white matter correlating with HIV encephalopathy. The other two cases had no specific findings.

**Table 2:** Postmortem macroscopic and neurohistologic findings in initial prospective IPV case series

Case	Age (decade)	Neck compression	Torso/extremity injury			Neuropathology										
				Ex vivo imaging	Brain weight (g)	TBI	DAI	WM microglia/ macrophages	Perivascular/ parenchymal iron	Vascular disease	CTE-NC	AD-NC	LBD	ARTAG	CAA	Other
NY1	3rd	–	R	–	1100^a^	R	–	+	–	–	–	–	–	+	–	Slight chronic meningitis
NY2	3rd	–	–	+	1010^a^	O	–	–	–	Old cortical infarct^b^	–	–	–	–	–	HIVE and leukoencephalopathy^b^
NY3	3rd	–	O	+	1318	–	+	–	+	–	–	–	–	–	–	–
NY4	4th	O	O/R	–	1300	O	–	+	+	–	–	–	–	–	–	Cortical microdysgenesis; possible PART
NY5	4th	–	R	–	1170^a^	R	+	+	+	–	–	–	–	–	–	Possible PART
NY6	4th	–	O	+	1330	O/R	+	+	+	Perivascular edema and mineralization in WM^b^	–	–	–	–	–	–
NY7	4th	O/R	R	–	1380	R	–	+	+	–	–	–	–	–	–	–
NY8	4th	R	R	+	1352	O	+	+	+	–	–	–	–	–	–	–
NY9	5th	–	R	–	1350	–	+	+	+	Lacune	–	–	–	+	–	
NY10	5th	–	–	–	950^a^	–	+	+	+	–	–	–	–	–	–	Slight chronic meningitis
NY11	6th	–	–	–	1360	O/R	+	+	+	Old and organizing infarcts; acute ischemia	–	–	–	–	–	–
NY12	6th	–	–	–	1300	O/R	+	+	+	Lacune; old cortical infarct	–	–	–	–	–	Mesial temporal sclerosis
NY13	7th	–	R	–	1150^a^	O	+	+	+	Lacune	–	–	–	–	–	–
NY14	8th	–	–	–	900^a^	O	–	+	–	Old cortical microinfarct	–	Low	–	+	+	Remote temporal lobectomy for seizures

+  assessed and present, – assessed and not present, *AD-NC* Alzheimer Disease neuropathologic change, ^*24*^*ARTAG* “age-related” tau astrogliopathy, *CAA* cerebral amyloid angiopathy, *CTE* chronic traumatic encephalopathy, *DAI* diffuse (traumatic) axonal injury, *HIVE* human immunodeficiency virus encephalitis, *HS* hippocampal sclerosis of aging, *NA* data not assessed/available, *NY#* from New York City Office of Chief Medical Examiner, *PART* Primary “age-related” tauopathy, *O* old, *R* recent/acute, *SDH* subdural hematoma, *TBI* traumatic brain injury, *WM* white matter

^a^Atrophy (brain weight less than expected [1200–1500 g])

^b^Detected on ex vivo imaging

See text for further explanation and definitions used, and figures for examples. See Supplement for details of findings in archival cohorts

#### Postmortem and neuropathologic findings

Macroscopic postmortem findings of trauma comprised acute and older visible bruising of face, extremities, torso, and scalp (seen in 8 cases) (Table 2). Old fractures of neck cartilages were found in 2, corroborating the histories of nonfatal strangulation; 1 of these cases plus one other case also had neck soft-tissue hemorrhage indicative of significant acute compression. Past evidence of TBI (e.g., healed cortical contusions, old subdural neomembranes) was documented in 8. Fenestration of the septum pellucidum was present in one. Recent focal TBI pathologies such as subdural and/or subarachnoid hemorrhage and contusions were present in 6. Of note, 6 had brain weights lower than expected for age [61].

**Fig. 2:**
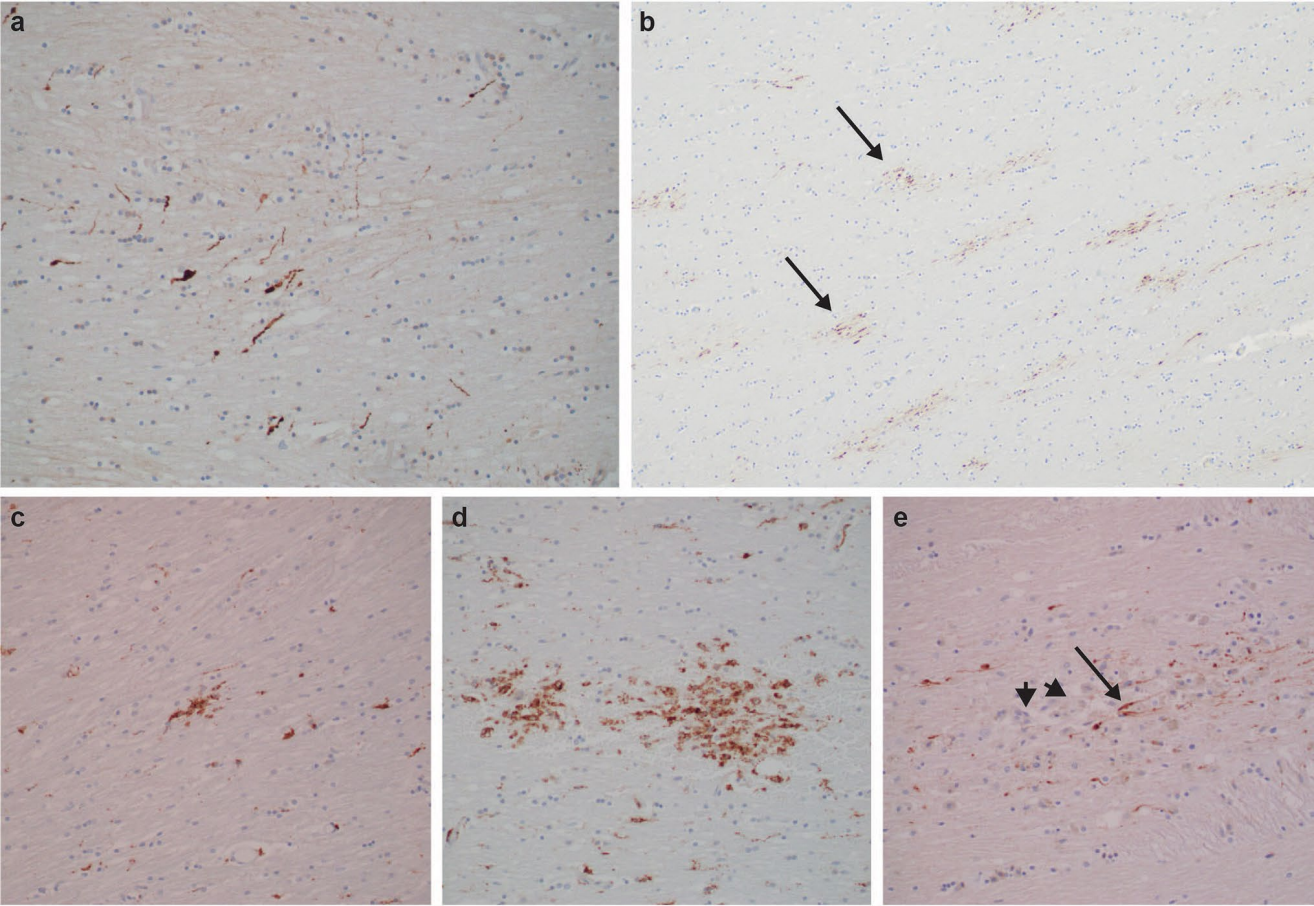
Histopathologic evidence of recent and old diffuse axonal injury in IPV. APP immunohistochemistry (brown reaction product) showing loose groupings (**a**., 200x; NY9, corpus callosum), or many clusters (arrows) of beaded axons (**b**., 100x; NY6, corpus callosum), indicating recent traumatic axonal injury, distinct from sparse staining in normal controls (not shown) or from the “zigzag” staining pattern expected in ischemic damage (not shown). CD68 immunostains highlighting single small (**c**., 200x; NY12, posterior limb of internal capsule) or larger grouped (**d**., 200x; NY6, corpus callosum) clusters of microglia and macrophages, considered markers of prior diffuse axonal injury. Microscopic focus of old, organizing diffuse axonal injury as detected by APP-immunopositive linear swollen axons (arrow), amid many unstained macrophages (arrowheads) (**e**., 200x; NY11, internal capsule). See text for discussion

Microscopically, in sections of the corpus callosum, internal capsule, and dorsolateral brainstem, in 9 cases, we identified APP-immunoreactive axons that were entirely consistent with stereotypical descriptions of acute diffuse axonal injury (DAI) in terms of their morphology, pattern, and distribution [26, 44] (Fig. 2). Over and above acute DAI, clusters of CD68-immunoreactive microglia were present in 12/14 cases, with or without axonal swellings, consistent with post-TBI associated ongoing neuroinflammation and axonal degeneration as previously described (Fig. 2) (see Discussion) [25].

**Fig. 3:**
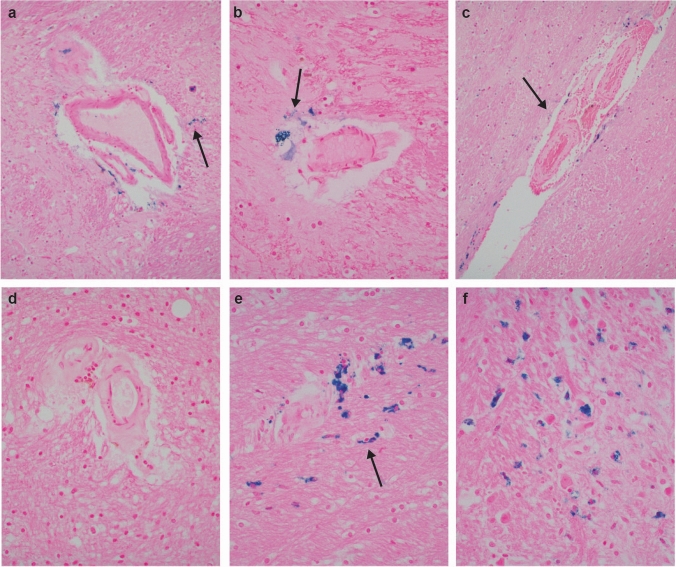
Histopathologic evidence of old vascular injury in IPV. Iron stains highlighting deposition of iron (blue granules; arrows) free and in macrophages in neuropil around non-arteriolosclerotic blood vessels in basal ganglia (**a**., 200x; NY10) and hypothalamus (**b**., 400x; NY7). Microvessels with arteriolosclerosis denoted by hyaline thickening of walls, and with iron in perivascular neuropil (arrow in **c**., 200x; NY5, posterior limb of internal capsule), in contrast to an arteriolosclerotic vessel with no iron in perivascular space or parenchyma (**d**., 400x; NY13). Old microbleeds detected by iron stains in neuropil (arrow) at sites vulnerable to traumatic axonal injury, such as the midbrain (**e**., around arteriolosclerotic vessel) and pons (**f**.; both, 400x; NY11). See text for discussion

Elsewhere, cerebral microvessel abnormalities were detailed as either: (1) typical for arteriolosclerotic (hypertensive, small vessel) disease (i.e., with hyaline mural thickening, mural or perivascular macrophages containing pigment, and perivascular rarefaction of neuropil); or (2) foci of perivascular/parenchymal Perls’ Prussian blue-positive iron deposition, without hyaline mural thickening. Examples of the former process, marked by lacunar infarcts, were noted in 3 cases. By contrast, the latter pattern was detected in 9 of the prospective 14, in 2 to a marked degree (Fig. 3) (see Discussion). Cerebral cortical infarcts were identified in 4, of which 3 were older women with known cardiac disease and pacemakers.

Only the oldest case (eighth decade of life) had evidence of Alzheimer Disease neuropathologic change and cerebral amyloid angiopathy. Regarding other abnormal tau deposition, "aging-related" tau astrogliopathy (ARTAG) was seen in 3 cases, 2 only in their third and fifth decades of life. Slight tau in neurites or rare neurons was detected in the neocortex in 4, hypothalamus in 3, hippocampus in 2 (both in fourth decade, possibly representing primary "age-related" tauopathy), amygdala in 2, and brainstem in 1. None met pathognomonic or supportive criteria for the diagnosis of CTE-NC. Lewy body pathology was absent.

### Secondary archival case series

These cases comprise archival IPV sets from the CONNECT-TBI study and from the Province of Manitoba. The former collection (*n* = 9; age range 3rd–9th decade; median, 6th) was notable for macroscopic postmortem findings of recent torso or extremity trauma in 4 cases; soft-tissue hemorrhage of the neck indicative of significant acute compression in 2; macroscopic evidence of old TBI (as characterized above) in 1, and of recent TBI in 4. Microscopic white matter microglia/macrophages (i.e., marker of prior TBI, though not specific to TBI) were noted in the one case for which this stain was available, and arteriolosclerosis in one. One case had ARTAG. Two cases, both in 7th decade or older, showed high Alzheimer’s disease neuropathologic change (AD-NC) and cerebral amyloid angiopathy (Supplementary Table 1). The Province of Manitoba archival set (*n* = 61; age range 2nd–9th decade (median, 4th) had clinical history of depression in 6; epilepsy in 3; and alcohol use disorder in 31 (combined with other substance use in 19). Macroscopically, 15 cases had acute TBI and 4 had old subdural hemorrhage; 7 had acute hypoxic-ischemic brain damage related to the circumstances of death; and 1 had old hypoglycemic brain damage. None had old contusions. Twenty-nine cases had comprehensive microscopic examination, including a full CTE workup, while the remainder had tau immunostaining of hippocampus and frontal cortex. With respect to tau pathology, 45 had no abnormality (median age, 3^rd^ decade); 12 had rare neurofibrillary tangles and neurites, mainly in the hippocampus and parahippocampal region (median age, 5th decade); 1 had ARTAG; and 2 had AD-NC, one low and one high (7th and 9th decade, respectively) (Supplementary Table 2). No CTE neuropathologic change or Lewy body pathology was found in either archival case series.

## Discussion

Despite the magnitude of the problem posed by physical intimate partner violence—the prevalence of which toward women is estimated at between 33 and 61% [23, 51]—the empirical investigation of IPV-related brain injury has been conspicuously sparse in the medical literature. It was our aim to begin the process of systematic case analyses of the brains of IPV victims, by crucial collaboration among academic centers and medical examiner/coroner settings under whose jurisdictions these victims fall. Our data indicate that such partnership can be successful and can begin to delineate the specific research questions for hypothesis generation in this population.

While TBI was detected grossly or microscopically in all 14 women with IPV in our prospectively accrued group, none of them had CTE-NC on comprehensive neuropathologic analysis. Likewise, in an additional convenience sample of 70 women with IPV, many had appreciable old or recent trauma, but only a few had minor age-related proteinopathies, none meeting consensus criteria for CTE-NC (see discussion of limitations, below). Our combined case series was notable for low brain weight suggestive of diffuse atrophy [24].

The finding of “age-related” tau astrogliopathy (ARTAG) in two relatively young subjects (3^rd^ and 5^th^ decades) in the prospective case series is of uncertain significance. Notably, our group’s prior observations that glial tau *enhanced* the detection of CTE-NC lent reassurance to us that we were not “missing” that diagnosis [2], and did not trigger additional sampling. Regarding the suggestion of primary “age-related” tauopathy (PART) in two subjects (4^th^ decade), this observation is not in itself outside of published experience [9]. The potential relationship of ARTAG or PART to TBI is an active topic of study in the field, and cannot be addressed meaningfully in this study.

In addition to the absence of CTE-NC in our case series, we were struck by microscopic findings which, although subtle, suggest old and recent traumatic axonal and vascular injury. These changes may reflect both mechanical (impact or torsional) injury as well as hypoxic-ischemic factors, such as may result from nonfatal strangulation (NFS).

With respect to NFS, some estimates suggest 3–10% of women in the general population [55], and 27–68% of IPV survivors have experienced it at least once [28]. Of relevance to this type of injury exposure, reported neuropathologic examination of 36 hanging deaths highlighted two key phenomena: “perivascular (perivenular) edematous neuropil” (resembling the vascular changes detected by ex vivo neuroimaging and histology, illustrated in Fig. 1), and “congestive bleeding around a venule” [52], which could be visible at later timepoints as iron deposition around small vessels. Thus, our observations of perivascular Perls’ Prussian blue-positive iron deposition (in most instances *not* associated with hypertensive vascular changes) among our prospective subjects beg the question of a role for NFS in the brain dysfunction in IPV survivors. This speculation will of course require larger case–control studies, since it is known that at least *coronary* vascular disease is prevalent among women experiencing relationship distress and violence [22, 59]. Of note, recent work also suggests microvascular compromise among individuals with repetitive head impacts [27].

Ex vivo MRI, though performed in only a subset of our series, proved particularly useful in identifying lesions occult to standard sampling [18] and in permitting more robust characterization of some vascular pathologies. Its use in future studies among deceased subjects will be of interest, and of potential import to the analysis of living IPV victims.

Regarding neuropathologic evidence of torsional brain injury (with or without impact), our focus was on well-characterized features published in subjects with known survivals after TBI, namely, microscopic collections of macrophages, with and without APP-immunoreactive axons or iron deposition (i.e., old traumatic hemorrhagic tissue tears) in white matter sites known to be susceptible to DAI [25]. These were readily identified among our prospective cases, and lacked an alternative explanation (e.g., infection, lacunar infarction), pointing toward a hypothesis to be tested among larger cohorts.

Finally, despite lack of some clinical data (including tobacco use) in our cohorts, the prevalence of seizures, asthma, HIV, substance use [4, 41], and other medical conditions known to contribute to vascular brain injury is striking. Elevated rates of multi-system chronic disease have been reported across multiple studies of IPV survivors, and marginalized individuals are at elevated risk for both IPV [38, 54] and health care inequity [19]. We speculate whether our findings may reflect the cumulative effects of prolonged IPV alongside poorly managed chronic disease.

### Limitations

Sampling bias must be carefully considered when interpreting the current findings. IPV cases that come through forensic settings may differ from individuals with incidental IPV histories identified in neurodegenerative disease banks [10]. Those whose deaths are related to IPV may be disproportionately marginalized and disconnected from health care, and their younger age at death could imply either more severe IPV, shorter duration of exposure to IPV, or both. The absence of CTE neuropathologic change observed across the initial prospective and secondary case series studied herein could reflect a true paucity of CTE in this population (e.g., if the frequency and intensity of IPV-related brain trauma is inconsistent with that required to develop CTE [58]), a biological difference in response to repetitive head trauma among women [20], poor case ascertainment (i.e., women with longstanding RHI secondary to IPV not identified through available information), the young median age of this cohort, or suboptimal tissue sampling among some cases in the archival series [7]. Finally, unavailability of clinical data among potential “control” women decedents precluded inclusion of an appropriate comparative sample.

## Conclusions

Data from the current series, reflecting close cooperation among forensic and academic centers, provide evidence of appreciable organic neurological disease, though conspicuously *not* CTE-NC, in women with IPV. Notably, our findings highlight the complex and likely contributory confounders of alcohol and/or substance use, epilepsy, HIV infection, and systemic vascular disease in this marginalized and highly vulnerable population. Larger investigations in a prospective IPV cohort, ideally leveraging some yet-to-be-defined appropriate control population, will provide the opportunity to advance our understanding of the clinical phenotypes and pathological substrates of multiple-etiology dementia [1]. Perhaps most importantly, with increased societal awareness, the consequences of IPV may be preventable and/or treatable with screening [15, 16] and life-saving intervention [5, 6, 37, 49].

### Supplementary Information

Below is the link to the electronic supplementary material.

Supplementary file1 (DOCX 4693 kb)

## Data Availability

The data supporting the conclusions of this article are included within the article and associated Supplementary file.

## References

[1] Rost NS (2022) Alzheimer's Disease-Related Dementias Summit 2022. NIH National Institute of Neurological Disorders and Stroke

[2] Ameen-Ali KE, Bretzin A, Lee EB, Folkerth R, Hazrati LN, Iacono D (2022). Detection of astrocytic tau pathology facilitates recognition of chronic traumatic encephalopathy neuropathologic change. Acta Neuropathol Commun.

[3] American Congress of Rehabilitation M (1993). Definition of mild traumatic brain injury. J Head Trauma Rehabil.

[4] Asare BY, Agyemang-Duah W, Adomako EB, Puri P, Ogundare DO, Vishwakarma D (2022). Association between experiences of intimate partner sexual violence and cigarette smoking among women in union in Papua New Guinea: evidence from a nationally representative survey. BMC Public Health.

[5] Bair-Merritt MH, Lewis-O'Connor A, Goel S, Amato P, Ismailji T, Jelley M (2014). Primary care-based interventions for intimate partner violence: a systematic review. Am J Prev Med.

[6] Bergin A, Blumenfeld E, Anderson JC, Campbell JC, Patch M (2022). Describing nonfatal intimate partner strangulation presentation and evaluation in a community-based hospital: partnerships between the emergency department and in-house advocates. J Head Trauma Rehabil.

[7] Bieniek KF, Cairns NJ, Crary JF, Dickson DW, Folkerth RD, Keene CD (2021). The Second NINDS/NIBIB consensus meeting to define neuropathological criteria for the diagnosis of chronic traumatic encephalopathy. J Neuropathol Exp Neurol.

[8] Bieniek KF, Ross OA, Cormier KA, Walton RL, Soto-Ortolaza A, Johnston AE (2015). Chronic traumatic encephalopathy pathology in a neurodegenerative disorders brain bank. Acta Neuropathol.

[9] Braak H, Thal DR, Ghebremedhin E, Del Tredici K (2011). Stages of the pathologic process in Alzheimer disease: age categories from 1 to 100 years. J Neuropathol Exp Neurol.

[10] Cations M, Keage HAD, Laver KE, Byles J, Loxton D (2022). Intimate partner violence and risk for mortality and incident dementia in older women. J Interpers Violence.

[11] Clark CJ, Alonso A, Everson-Rose SA, Spencer RA, Brady SS, Resnick MD (2016). Intimate partner violence in late adolescence and young adulthood and subsequent cardiovascular risk in adulthood. Prev Med.

[12] Clarot F, Vaz E, Papin F, Proust B (2005). Fatal and non-fatal bilateral delayed carotid artery dissection after manual strangulation. Forensic Sci Int.

[13] Corrigan JD, Yang J, Singichetti B, Manchester K, Bogner J (2018). Lifetime prevalence of traumatic brain injury with loss of consciousness. Inj Prev.

[14] Crane PK, Gibbons LE, Dams-O'Connor K, Trittschuh E, Leverenz JB, Keene CD (2016). Association of traumatic brain injury with late-life neurodegenerative conditions and neuropathologic findings. JAMA Neurol.

[15] Curry SJ, Krist AH, Owens DK, Barry MJ, Caughey AB, Davidson KW (2018). Screening for intimate partner violence, elder abuse, and abuse of vulnerable adults: us preventive services task force final recommendation statement. JAMA.

[16] Dams-O'Connor K, Bulas A, Lin Haag H, Spielman LA, Fernandez A, Frederick-Hawley L (2023). Screening for Brain Injury Sustained in the Context of Intimate Partner Violence (IPV): Measure Development and Preliminary Utility of the Brain Injury Screening Questionnaire IPV Module. J Neurotrauma:.

[17] Danielsen T, Hauch C, Kelly L, White CL (2021). Chronic traumatic encephalopathy (CTE)-type neuropathology in a young victim of domestic abuse. J Neuropathol Exp Neurol.

[18] Edlow BL, Keene CD, Perl DP, Iacono D, Folkerth RD, Stewart W (2018). Multimodal characterization of the late effects of traumatic brain injury: a methodological overview of the late effects of traumatic brain injury project. J Neurotrauma.

[19] Femi-Ajao O, Kendal S, Lovell K (2020). A qualitative systematic review of published work on disclosure and help-seeking for domestic violence and abuse among women from ethnic minority populations in the UK. Ethn Health.

[20] Ferguson SA, Mouzon BC, Lynch C, Lungmus C, Morin A, Crynen G (2017). Negative impact of female sex on outcomes from repetitive mild traumatic brain injury in hTau mice is age dependent: a chronic effects of neurotrauma consortium study. Front Aging Neurosci.

[21] Fowler D (2007). The extent of substance use problems among women partner abuse survivors residing in a domestic violence shelter. Fam Community Health.

[22] Gallo LC, Troxel WM, Kuller LH, Sutton-Tyrrell K, Edmundowicz D, Matthews KA (2003). Marital status, marital quality, and atherosclerotic burden in postmenopausal women. Psychosom Med.

[23] Garcia-Moreno C, Jansen HA, Ellsberg M, Heise L, Watts CH (2006). Prevalence of intimate partner violence: findings from the WHO multi-country study on women's health and domestic violence. Lancet.

[24] Gibbons LE, Power MC, Walker RL, Kumar RG, Murphy A, Latimer CS (2023). Association of traumatic brain injury with late life neuropathological outcomes in a community-based cohort. J Alzheimers Dis.

[25] Johnson VE, Stewart JE, Begbie FD, Trojanowski JQ, Smith DH, Stewart W (2013). Inflammation and white matter degeneration persist for years after a single traumatic brain injury. Brain.

[26] Johnson VE, Stewart W, Smith DH (2013). Axonal pathology in traumatic brain injury. Exp Neurol.

[27] Kirsch D, Shah A, Dixon E, Kelley H, Cherry JD, Xia W (2023). Vascular injury is associated with repetitive head impacts and tau pathology in chronic traumatic encephalopathy. J Neuropathol Exp Neurol.

[28] Kwako LE, Glass N, Campbell J, Melvin KC, Barr T, Gill JM (2011). Traumatic brain injury in intimate partner violence: a critical review of outcomes and mechanisms. Trauma Violence Abuse.

[29] Lee EB, Kennedy-Dietrich C, Geddes JF, Nicoll JAR, Revesz T, Smith DH, Stewart W (2023). The perils of contact sport: pathologies of diffuse brain swelling and chronic traumatic encephalopathy neuropathologic change in a 23-year-old rugby union player. Acta Neuropathol.

[30] Lee EB, Kinch K, Johnson VE, Trojanowski JQ, Smith DH, Stewart W (2019). Chronic traumatic encephalopathy is a common co-morbidity, but less frequent primary dementia in former soccer and rugby players. Acta Neuropathol.

[31] Lehman EJ, Hein MJ, Baron SL, Gersic CM (2012). Neurodegenerative causes of death among retired National Football League players. Neurology.

[32] Livingston G, Huntley J, Sommerlad A, Ames D, Ballard C, Banerjee S (2020). Dementia prevention, intervention, and care: 2020 report of the Lancet Commission. Lancet.

[33] Mackay DF, Russell ER, Stewart K, MacLean JA, Pell JP, Stewart W (2019). Neurodegenerative disease mortality among former professional soccer players. N Engl J Med.

[34] Malek AM, Higashida RT, Halbach VV, Dowd CF, Phatouros CC, Lempert TE (2000). Patient presentation, angiographic features, and treatment of strangulation-induced bilateral dissection of the cervical internal carotid artery. Report of three cases J Neurosurg.

[35] McCloskey LA, Williams CM, Lichter E, Gerber M, Ganz ML, Sege R (2007). Abused women disclose partner interference with health care: an unrecognized form of battering. J Gen Intern Med.

[36] McKee AC, Cairns NJ, Dickson DW, Folkerth RD, Dirk Keene C, Litvan I (2016). The first NINDS/NIBIB consensus meeting to define neuropathological criteria for the diagnosis of chronic traumatic encephalopathy. Acta Neuropathol.

[37] McKee GB, Gill-Hopple K, Oesterle DW, Daigle LE, Gilmore AK (2022). New perspectives on risk factors for non-fatal strangulation and post-assault imaging. J Interpers Violence.

[38] Messinger AM (2011). Invisible victims: same-sex IPV in the National Violence Against Women Survey. J Interpers Violence.

[39] Monahan K, Bannon S, Dams-O’Connor K (2020). Nonfatal strangulation (NFS) and intimate partner violence: A brief overview. J Fam Violence.

[40] Montine TJ, Phelps CH, Beach TG, Bigio EH, Cairns NJ, Dickson DW (2012). National Institute on Aging-Alzheimer's Association guidelines for the neuropathologic assessment of Alzheimer's disease: a practical approach. Acta Neuropathol.

[41] Nemeth JM, Bonomi AE, Lu B, Lomax RG, Wewers ME (2016). Risk factors for smoking in rural women: the role of gender-based sexual and intimate partner violence. J Womens Health (Larchmt).

[42] Ng SY, Lee AYW (2019). Traumatic brain injuries: pathophysiology and potential therapeutic targets. Front Cell Neurosci.

[43] Phillips DY, Walsh B, Bullion JW, Reid PV, Bacon K, Okoro N (2014). The intersection of intimate partner violence and HIV in U.S. women: a review. J Assoc Nurses AIDS Care.

[44] Reichard RR, Smith C, Graham DI (2005). The significance of beta-APP immunoreactivity in forensic practice. Neuropathol Appl Neurobiol.

[45] Renner LM, Spencer RA, Morrissette J, Lewis-Dmello A, Michel H, Anders D (2021). Implications of severe polyvictimization for cardiovascular disease risk among female survivors of violence. J Interpers Violence.

[46] Research FITBI Chronic TBI-Related Neurodgeneration Common Data Elements https://fitbir.nih.gov/chronic-tbi-related-neurodegeneration-cdes. Accessed 05 Jan 2023

[47] Roberts GW, Whitwell HL, Acland PR, Bruton CJ (1990). Dementia in a punch-drunk wife. Lancet.

[48] Russell ER, Mackay DF, Lyall D, Stewart K, MacLean JA, Robson J (2022). Neurodegenerative disease risk among former international rugby union players. J Neurol Neurosurg Psychiatry.

[49] Sampsel K (2021). Commentary on underrecognition of nonfatal intimate partner strangulation within the emergency health care setting: reasons and opportunities for change. J Emerg Nurs.

[50] Sandsmark DK, Bashir A, Wellington CL, Diaz-Arrastia R (2019). Cerebral microvascular injury: a potentially treatable endophenotype of traumatic brain injury-induced neurodegeneration. Neuron.

[51] Sardinha L, Maheu-Giroux M, Stöckl H, Meyer SR, García-Moreno C (2022). Global, regional, and national prevalence estimates of physical or sexual, or both, intimate partner violence against women in 2018. Lancet.

[52] Schröder R, Saternus KS (1983). Congestion in the area of the head and changes in the brain caused by suicidal hanging death. Z Rechtsmed.

[53] Smith DH, Dollé JP, Ameen-Ali KE, Bretzin A, Cortes E, Crary JF (2021). COllaborative Neuropathology NEtwork Characterizing ouTcomes of TBI (CONNECT-TBI). Acta Neuropathol Commun.

[54] Smith S, Chen J, Basile K, Gilbert L, Merrick M, Patel N, Walling M, Jain A (2017) The National Intimate Partner and Sexual Violence Survey (NISVS): 2010–2012 State Report.

[55] Sorenson SB, Joshi M, Sivitz E (2014). A systematic review of the epidemiology of nonfatal strangulation, a human rights and health concern. Am J Public Health.

[56] St Ivany A, Schminkey D (2016). Intimate partner violence and traumatic brain injury: state of the science and next steps. Fam Community Health.

[57] Stram MN, Seifert AC, Cortes E, Akyatan A, Woodoff-Leith E, Borukhov V (2022). Neuropathology of pediatric SARS-CoV-2 infection in the forensic setting: novel application of ex vivo imaging in analysis of brain microvasculature. Front Neurol.

[58] VanItallie TB (2019). Traumatic brain injury (TBI) in collision sports: Possible mechanisms of transformation into chronic traumatic encephalopathy (CTE). Metabolism.

[59] Wang HX, Leineweber C, Kirkeeide R, Svane B, Schenck-Gustafsson K, Theorell T (2007). Psychosocial stress and atherosclerosis: family and work stress accelerate progression of coronary disease in women. The Stockholm Female Coronary Angiography Study. J Intern Med.

[60] Whiteneck GG, Cuthbert JP, Corrigan JD, Bogner JA (2016). Prevalence of self-reported lifetime history of traumatic brain injury and associated disability: a statewide population-based survey. J Head Trauma Rehabil.

[61] Whitwell H, Milroy C, du Plessis D (2021). Forensic neuropathology.

[62] Wu XM, Zhang XD, Yun LB, Liu M, Yi XF (2017). Sudden death from ruptured intracranial vascular malformations during mechanical asphyxia: a domestic violence case report. Am J Forensic Med Pathol.

[63] Zieman G, Bridwell A, Cardenas JF (2017). Traumatic brain injury in domestic violence victims: a retrospective study at the Barrow Neurological Institute. J Neurotrauma.

