# Supplementary material for: The neuropathology of intimate partner violence
Source: Acta Neuropathol. 2023 Oct 28;146(6):803–15. doi: 10.1007/s00401-023-02646-1 (PMC10627910; doi:10.1007/s00401-023-02646-1)
Supplement: Supplementary file 1 — Supplementary file1 (DOCX 4693 kb) [file 401_2023_2646_MOESM1_ESM.docx]

Autopsy Neuropathology of Intimate Partner Violence (IPV): A Case Series

Supplement Elements Include:

- Table 1 - Clinical, Autopsy, and Neuropathology Findings, CONNECT-TBI Archival Cohort
- Table 2 - Clinical, Autopsy, and Neuropathology Findings, Province of Manitoba Archival Cohort
- Figure 1

**Details of CONNECT-TBI Archival Cohort**

**METHODS**

Cases were obtained retrospectively from the virtual holdings of the CONNECT-TBI collaborative group [23]. The group (a “center without walls”) serves as a repository for annotated cases from its component institutions, for which data and tissues are available to the larger traumatic brain injury (TBI) community.

Search of the holdings performed in summer, 2022, detected 9 suitable cases with documented histories of IPV, per harmonized clinical data associated with each specimen, all having undergone complete neuropathologic examination, including sampling and immunostaining for CTE (see Methods in main manuscript). Of note, as archival cases, these were not available for the *ex vivo* neuroimaging studies, nor the additional histologic studies for old/recent traumatic axonal injury. Even so, 2 of 9 cases had some assessment for DAI (using axonal and/or macrophage/microglial markers). All had ascertainment of cerebrovascular disease by the submitting pathologist.

**RESULTS**

These are summarized in the manuscript, with details in Table 1.

Supplement Table 1. Clinical, Autopsy, and Neuropathology Findings, CONNECT-TBI Archival Cohort

| **Case** | **Age (decade)** | **Neck com-pression** | **Torso/**  **extremity injury** |  | **Neuropathology** | | | | | | | | | | |
| --- | --- | --- | --- | --- | --- | --- | --- | --- | --- | --- | --- | --- | --- | --- | --- |
|  |  |  |  | **Brain weight (g)** | **TBI** | **DAI** | **WM microglia/ macrophages** | **Perivascular/ parenchymal iron** | **Vascular disease** | **CTE-NC** | **AD-NC** | **LBD** | **ARTAG** | **CAA** | **Other** |
| CN1 | 3^rd^ | - | R | 1360 | R | NA | NA | NA | - | - | - | - | - | - | Acute ischemia |
| CN2 | 4^th^ | - | R | 1000* | R | + | NA | NA | - | - | - | - | - | - | - |
| CN3 | 5^th^ | R | R | 1236 | R | NA | NA | NA | - | - | - | - | - | - | Prior SDH evacuation |
| CN4 | 5^th^ | - | - | 1450 | - | NA | + | NA | - | - | - | - | - | - | - |
| CN5 | 6^th^ | - | - | 1457 | - | NA | NA | NA | - | - | - | - | - | - | Meningioma, CNS WHO grade 1 |
| CN6 | 6^th^ | - | - | 1280 | - | NA | NA | NA | - | - | - | - | - | - | - |
| CN7 | 6^th^ | R | R | 1250 | O/R | + | NA | NA | - | - | - | - | - | - | PART; acute ischemia |
| CN8 | 7^th^ | - | - | 1293 | - | NA | NA | NA | Lacunes | - | high | - | - | + | - |
| CN9 | 9^th^ | - | - | 1016* | - | NA | NA | NA | - | - | high | - | + | + | FTLD-TDP, type A; HS |

Table 1 Legend: +=assessed and present; -=assessed and evidence not found; *=atrophy (brain weight less than expected [1200-1500g]); AD-NC=Alzheimer Disease neuropathologic change;^24^ ARTAG=“age-related” tau astrogliopathy; CAA= cerebral amyloid angiopathy; CN#=CONNECT-TBI case number; CNS WHO=central nervous system World Health Organization; CTE=chronic traumatic encephalopathy; DAI=traumatic axonal injury; FTLD-TDP=frontotemporal lobar degeneration-tar DNA-binding protein; HS=hippocampal sclerosis of aging; NA=data not assessed/available; PART=Primary “age-related” tauopathy; R=recent/acute; SDH=subdural hematoma; TBI=traumatic brain injury.

**Details of Province of Manitoba Archival Cohort**

**METHODS**

This retrospective cohort was assembled in Winnipeg, Manitoba, Canada in accordance with departmental tissue use guidelines and was approved by the Health Research Ethics Board at the University of Manitoba (protocol number HS23503 / H2019-504). In Manitoba, all medicolegal autopsies are conducted in two major teaching hospitals by a coordinated service of forensic autopsy pathologists. This includes approximately 1200 autopsies per year for a population of 1.4 million. All neuropathology consultation services are conducted in a single center, which allows true population-based studies. Death investigation in Manitoba is essentially similar to that described in the main text for Manhattan. In 2011 Manitoba had the second highest per capita rate of family violence among the provinces of Canada (402 police-reported violent offences against family members per 100,000 population per year) [22], and in 2019 Manitoba had the highest provincial rate of ‘femicide’ in Canada [8].

Autopsy records (2000-2022 inclusive) are searchable by free text using DocFetcher software(http://docfetcher.sourceforge.net/en/index.html). The Boolean operator search strategy was: “domestic OR abuse OR abusive OR maltreatment OR mistreat* OR molest* OR assault* OR violence OR stab* OR struck OR hit OR pushed OR punch* OR beat* OR strike* OR harm*” AND “spouse OR boyfriend OR common law OR husband OR partner”. Note that there were no hits for “family violence”, “abusive relationship”, “wife abuse”, “partner abuse/violence” and maltreatment/mistreatment. For this analysis, all cases are female.

In addition, the archives of the Winnipeg Free Press, a local newspaper that covers provincial matters (https://newspaperarchive.com/browse/ca/mb/winnipeg/winnipeg-free-press/) were searched using the words “homicide OR murder OR violence” AND “partner OR domestic”. Using the autopsy records, the medical imaging database, and the newspaper accounts (which were also searched for the autopsy victims’ names), the following information was sought: age at time of death, year of death, previous brain injuries, previous neurologic / psychiatric disease, brain imaging findings, substance use (alcohol, drugs), manner of death, cause of death, and brain abnormalities on neuropathologic examination.

Glass slides and paraffin blocks were retrieved from storage. Brain samples from autopsies without neuropathology consultation had been fixed for 2-5 days before embedding in paraffin. All had sampling of the hippocampus/entorhinal region and the frontal cortex with additional locations (e.g. cerebellum) at the discretion of the forensic pathologist. Brains referred for complete neuropathology consultation were fixed in 10% buffered formalin for 10–40 days. Those brains were sampled widely (typically 10–16 regions total) including the lateral frontal, paramedian frontal, hippocampus/medial temporal, thalamus, basal nuclei, midbrain, pons, and cerebellum. In the absence of a history of neurodegenerative disease, brain sampling was not optimized for detection of CTE [3];not all cases had the amygdala sampled and those without a history of alcohol / drug abuse did not necessarily have the mammillary bodies sampled. Histologic preparation was standard. Immunostaining for p-tau was performed on freshly cut sections using the AT8 mouse monoclonal antibody (MN1020, ThermoFisher; dilution 1/3000; detects tau phosphorylated at Ser199 + Ser202 + Thr205) using the Dako EnVision + Dual Link System-HRP followed by counterstaining with hematoxylin. The method is the same as that used in the previous study of CTE in community source autopsy brains [19]. The current definition of the CTE-associated p-tau lesion was “The pathognomonic lesion consists of p-tau aggregates in neurons, as [neurofibrillary tangles] and disordered neurites, in the presence or absence of p-tau immunoreactive astrocytes, around small vessels in an irregular pattern at the depths of the cortical sulci” [3], taking into consideration the slightly contentious debate about what constitutes a significant CTE lesion [10, 11, 21, 24]. Care was taken to distinguish p-tau deposits characteristic of age-related tau astrogliopathy (ARTAG) [13], early tau-only lesions [5], and primary age-related tauopathy (PART) [6] from CTE [1, 15, 17]. The approximate size, location, and pattern of p-tau deposits were recorded. All case reviews and tissue examinations were conducted by a single neuropathologist (M. Del Bigio)

**RESULTS**

During the period of interest (2000-2022 inclusive), 64 autopsies were performed on women who had a credible history of intimate partner violence that had been documented in the medical records or in reporting of the criminal court proceedings (Supplement Table 2). To preserve anonymity, ages are presented in 5-year cohorts and the cases are sorted by age, not timing of autopsy. The majority was White (30) with 18 North American indigenous, 1 east Asian, and 15 unspecified ethnicities. The age range was 17-83 years (median 34 years; mean ± standard deviation, 36.5 ± 14.5 years). Thirty-one cases had a history of alcohol abuse, often combined with other drugs. Ten had a history of psychiatric disorder. The majority of deaths were classified as homicides (29/64) and suicides (14/64). Most of the homicides were by blunt trauma (n = 5), sharp trauma (i.e. stabbing) (n = 13), or strangulation (n = 5). None were known to have suffered prior non-fatal strangulation or drowning. Most of the suicides were by hanging (n = 10) or drug overdose. Among the deaths of undetermined manner (14/60), 7/14 were drug or alcohol overdoses that could not be determined with certainty to be accidental or deliberate (i.e., suicidal). The perpetrator of intimate partner abuse was not always specified in the autopsy report but was often documented in the press coverage of homicide cases. The perpetrator was the husband / partner / boyfriend in 42 cases, son in 1 case, parent in 1 case, and unspecified in the remainder. Among the 29 homicides, the killer was also the chronic abuser in all but 1 case. Details of the chronic abuse were almost always vague (e.g., “multiple assaults”, “history of domestic violence”, etc.). Previous contact with the medical system was rare. Of note, details of prior, possibly minor, head injuries (if any) are unknown. Prior imaging of the face / head / brain / neck for inexplicit histories of trauma had been documented in 17 cases (1-5 computed tomography or magnetic resonance imaging scans in individual cases). Fractures of facial bones were detected in 4 cases, but none had traumatic changes in the brain documented by imaging. Imaging records of the 12 most remote deaths were not electronically searchable.

Among the 64 cases, brains were examined in 61 (2 not examined due to post-mortem changes, 1 because of fragmentation by shotgun wound). Twenty-nine of 61 were fixed for examination by a neuropathologist and the other 32 were sliced and sampled at the time of autopsy by the forensic pathologist. Fifteen cases had acute traumatic brain injuries and 7 had acute hypoxic-ischemic brain damage related to the circumstances of death. None had gross or microscopic features of old contusions. Excluding features of acute brain trauma or hypoxic-ischemic damage related to the cause of death, pre-existing structural brain abnormalities were present in 8/61 cases (resolved subdural hematoma, 4; old frontal infarct, 1; old hypoglycemic brain damage, 1; Alzheimer disease-type changes, 2). Except for the Alzheimer disease case with dementia, no brains had evidence of atrophy or ventriculomegaly. See Supplement Table 2 for details.

The number of sampled regions potentially vulnerable to CTE-specific pathology (i.e., cerebral cortex and hippocampus) was tallied. These were chosen for p-tau immunostaining. Among the most recent 32 cases (2012-2022), 0-7 (median 2.0; mean ± SEM 2.6 ± 0.3) regions of interest were available. Among the earlier 29 cases (2000-2012), 0-4 (median 1.5; mean ± SEM 1.8 ± 0.2) regions were available. This difference between the two temporal cohorts was statistically significant (p=0.0202; two-tailed Student t test). It is reflective of our changing practice in the neuropathological examination of forensic cases wherein the sampling for microscopy is now more extensive.

Abnormal p-tau immunolabeling was present in 15/61 cases. These included 13 cases with rare neurites and / or neurons (neurofibrillary tangles) in the CA1 sector of the hippocampal formation and / or the parahippocampal gyrus. Rare p-tau positive entorhinal neurons beginning in the third decade were previously described by Braak [5]. They might represent an early manifestation of PART [6]. A single case with patchy subpial glial p-tau immunoreactivity and very rare entorhinal neurons was considered to be an early form of ARTAG. One woman in mid-60s had early Alzheimer disease type changes (A2B1C1) and one elderly woman with dementia had advanced Alzheimer disease-type changes (NIA-AA score A3 B2 C3) [18]. Definitive features characteristic of CTE were not seen in any case although a single woman in her late 30s had a single cluster of p-tau immunoreactive neurons at the depth of a sulcus (Supplement Figure 1).

Supplement Table 2. Clinical, Autopsy, and Neuropathology Findings, Province of Manitoba Archival Cohort

| **Age group** | **History of domestic violence** | **Drugs / alcohol / psychiatric history** | **Cause of death and autopsy findings** | **Manner of death** | **Brain weight (g)** | **Neuropathology findings** | **Tau findings** |
| --- | --- | --- | --- | --- | --- | --- | --- |
| 16-20 | Multiple assaults; multiple CT scans of head | Cannabis, methamphetamine | Found dead, no evidence trauma | Undetermined | N/A | - | - |
| 16-20 | Multiple assaults with loss of consciousness | Anxiety, depression, prior Suicide attempt, headaches | Drug overdose | Suicide | 1420 | - | - |
| 16-20 | Boyfriend assault | Alcohol | Acute brain trauma | Homicide | 1565 | Acute bilateral SDH and contusions | - |
| 16-20 | Domestic assaults | Alcohol | Blunt trauma torso | Homicide | 1080 | - | - |
| 16-20 | Abuse by boyfriend | Alcohol | Stabbed; spine and facial fractures | Homicide | 1260 | - | - |
| 16-20 | Beaten by common-law partner who “admitted to slapping her many times with full force” | Alcohol, cocaine | Acute brain and abdominal trauma | Homicide | 1245 | Resolved SDH; acute large SDH with herniations | - |
| 16-20 | Physical abuse as child | Alcohol, cocaine, oxycodone, morphine; personality disorder | Mixed drug toxicity | Undetermined | 1460 | - | - |
| 16-20 | Physical and sexual abuse by caregivers | Cocaine | Hanging | Suicide | 1360 | - | - |
| 16-20 | Previous assault then strangled by ex-boyfriend | N/A | Strangled | Homicide | 1285 | - | - |
| 21-25 | Boyfriend admitted to hacking to death with knife | N/A | Multiple stabbing torso | Homicide | 1390 | - | - |
| 21-25 | Domestic abuse | Alcohol, drugs NOS, gasoline sniffing; depression, | Hanging | Suicide | 1300 | - | - |
| 21-25 | Domestic abuse | Alcohol, drugs NOS | Hanging | Suicide | 1395 | - | - |
| 21-25 | Husband beat with hammer | N/A | Acute brain trauma | Homicide | 1385 | Skull fractures, contusions, SAH | - |
| 21-25 | Assaulted by common-law husband | Alcohol | Blunt trauma to head and hypothermia | Homicide | 1280 | Mild swelling | - |
| 21-25 | Previously abusive partner | Alcohol | Hanging | Suicide | 1285 | Resolved small SAH frontal | - |
| 26-30 | Domestic violence, multiple CT scans of head | N/A | Cardiomyopathy with arrythmia | Natural | 1485 | Hippocampal neuron loss secondary to hypoglycemic brain damage 1 year before death | - |
| 26-30 | Sexual and physical abuse (stabbed) | Alcohol; cocaine, amphetamine | Pedestrian MVA with multiple trauma | Accident | N/A | Skull fracture, brain fragmented | - |
| 26-30 | “Domestic partner ... horrifying incident of brutality” | Alcohol | Blunt trauma brain | Homicide | 1715 | Large SDH; old SDH bilateral | - |
| 26-30 | Pushed down stairs by husband | Alcohol | Minor head trauma | Undetermined | 1575 | - | Rare NFT, entorhinal and hippo CA2 and CA1 |
| 26-30 | Domestic violence | Alcohol | Stabbed in chest, multiple contusions | Homicide | 1365 | - | Rare NFT, entorhinal, parahippocampal;one focus neurons and neurites (but not perivascular or depth of sulcus); hippocampus - |
| 26-30 | Murder/Suicide - boyfriend | N/A | Multiple stab wounds | Homicide | N/A | - | - |
| 26-30 | Domestic abuse | N/A | Hanging; contusions face and limbs | Suicide | 1205 | - | - |
| 26-30 | Domestic violence | Alcohol | Acute ethanol toxicity | Undetermined | 1205 | - | - |
| 26-30 | Assaulted by husband 2 days earlier | N/A | Glyburide overdose, coma 2 days; minor contusions face and limbs | Suicide | 1223 | Acute severe HI/hypoglycemic damage with infarcts and swelling | - |
| 26-30 | Common-law husband | N/A | Stabbed + beating | Homicide | 1205 | - | - |
| 26-30 | Past physical abuse | Cocaine, alcohol | Fall from height | Suicide | 1410 | Small SAH; HI damage | - |
| 31-35 | Known domestic abuse | N/A | Blunt head trauma | Homicide | 1494 | Basal skull fracture, acute contusions | - |
| 31-35 | Assaulted by husband 2 days prior; seizure | N/A | Cardiac arrest after seizure; coma x4 days | Undetermined | 1480 | Subacute SDH, severe HI | - |
| 31-35 | Previous assaults by husband | N/A | All ribs fractured, mild head trauma | Homicide | 1404 | Minor SAH | - |
| 31-35 | Stabbed by common-law husband | Alcohol | Stabbed | Homicide | N/A | - | - |
| 31-35 | Husband psychotic | N/A | Multiple stab wounds | Homicide | 1335 | - | - |
| 31-35 | Domestic abuse | Alcohol | Acute ethanol toxicity; multiple contusions on limbs | Undetermined | 1230 | - | - |
| 31-35 | Assaulted by boyfriend | Alcohol, drugs NOS | Hanging | Suicide | 1320 | - | - |
| 36-40 | Stabbed in chest years ago; assaulted (concussion) 2 days prior to death | Alcohol | Recent injuries to face and torso | Undetermined | 1463 | Old small SDH | - |
| 36-40 | History of domestic violence with ex-partner, multiple CT scans of head | Alcohol, drugs NOS; depression with suicidal ideation | Hanging; multiple bruises | Suicide | 1225 | - | Rare NFT, depth frontal sulcus (but not characteristic of CTE), CA1, and scattered in parahippocampal gyrus |
| 36-40 | Previous domestic complaints | Alcohol & cocaine; previous Suicide attempts | Hanging | Suicide | 1375 | - | - |
| 36-40 | Boyfriend | Cocaine | Stabbed in chest; facial contusions | Homicide | 1250 | - | - |
| 36-40 | Boyfriend physical abuse | N/A | Stabbed | Homicide | N/A | - | - |
| 36-40 | Previous history of domestic abuse | Cocaine, benzodiazepines | Cocaine toxicity; cardiac arrest with resuscitation | Undetermined | 1290 | Acute HI encephalopathy | - |
| 36-40 | Pushed by common-law husband | Alcohol | Acute brain injury and alcohol toxicity | Undetermined | 1380 | Acute SAH, IVH (small) | - |
| 41-45 | Vague history of domestic violence | N/A | No anatomical cause | Undetermined | N/A | - | Very rare NFT, parahippocampal |
| 41-45 | Assaulted by boyfriend multiple times including 1 week prior to death | Alcohol, opioids | Possible strangulation, multiple contusions | Undetermined | 1195* | - | - |
| 41-45 | Previous recent domestic assaults | Alcohol; anorexia | Blunt head trauma (fall down stairs) + 10 day survival | Accident | 1545 | Skull fracture, contusions, SDH, ICH, HI, herniations | Very rare NFT, entorhinal |
| 41-45 | Multiple police calls for domestic fights | Alcohol, morphine, methamphetamine | Methamphetamine overdose with restraint; multiple bruises | Undetermined | 1220 | - | - |
| 41-45 | Domestic disputes husband | Cannabis | hanging | Suicide | 1280 | - | - |
| 41-45 | Domestic violence | Alcohol | stabbed + blunt head trauma | Homicide | 1205 | Skull fracture, acute patchy SAH, small contusions | - |
| 46-50 | Physical abuse; occasional seizures | Opioids / methadone; depression | pneumonia, sepsis, HIE | Natural | N/A | - | Rare NFT, CA1 and parahippocampal |
| 46-50 | Chronic domestic violence | N/A | Strangulation | Homicide | 1523 | Early HI | Very rare neurites, medial temporal cortex |
| 46-50 | Husband - murder Suicide; >20 years domestic disputes; | N/A | Stabbed | Homicide | N/A | - | - |
| 46-50 | Shot by husband - no known previous assault | N/A | Shotgun blast to chest | Homicide | 1405 | - | - |
| 51-55 | Domestic violence, “Several previous closed head injuries” headache, multiple MRI of head | N/A | beating - acute head/face trauma (survive 2 days) | Homicide | 1435 | Skull fracture, bilateral SDH, SAH, IVH, severe HI | - |
| 51-55 | Domestic abuse | N/A | Strangulation; multiple bruises | Homicide | 1250 | - | Rare NFT, entorhinal |
| 51-55 | Spousal abuse; multiple CT scans of head | Alcohol; seizure disorder and depression | Mixed drug toxicity; contusions on arms | Undetermined | 1190* | Early HI | Rare NFT, entorhinal |
| 51-55 | Stabbed by common-law husband | N/A | Stabbed | Homicide | 1250 | - | Rare NFT, entorhinal |
| 56-60 | History of domestic violence; repetitive facial injuries; multiple CT scans of head | Alcohol | Coronary artery disease with cardiac arrest; multiple bruises | Natural | 1266 | - | Rare NFT, CA1 |
| 56-60 | Assaulted by husband; no known prior history of domestic violence | N/A | Blunt head trauma | Homicide | N/A | - | - |
| 61-65 | Stabbed and beaten by husband; unknown if there had been prior assaults | N/A | Stabbed in face / head, multiple contusions | Homicide | N/A | - | Rare NFT, CA1 |
| 61-65 | Son abusive | Alcohol | Hanging; multiple bruises | Suicide | 1312 | Early Alzheimer-type changes A2B1C1; no cerebrovascular disease | NFT, medial temporal including hippocampus (Braak stage 2) |
| 61-65 | Domestic abuse | Oxycodone | Oxycodone toxicity | Undetermined | 1290 | - | Subpial neurites and rare NFT (ARTAG / PART) |
| 71-75 | Spousal abuse | N/A | Acute myocardial infarction; lymphoma | Natural | 1120* | SDH, recent small; old small frontal infarct | - |
| 81-85 | Dementia; stabbed by husband, no prior history of violence | N/A | Stabbed | Homicide | 1240 | Moderate patchy atheroma; Alzheimer-type pathology BAMY cortex, putamen, cerebellum including vessels (A3B2C3) | Rare NFT, frontal, entorhinal and hippocampus (Braak stage 3) |

Table 2 Legend:

-=assessed and not present; *=atrophy (brain weight less than expected [1200-1500g]); ARTAG=“age-related” tau astrogliopathy; BAMY=beta amyloid precursor protein; HI=hypoxic-ischemic type changes; IVH=intraventricular hemorrhage; MVA=motor vehicle accident; N/A=not available; NFT=neurofibrillary tangles; PART=primary “age-related” tauopathy; SAH=subarachnoid hemorrhage; SDH=subdural hemorrhage.

Supplement Figure 1.

Phosphorylated tau-immunoreactive neurons at depth of frontal lobe sulcus from a late 30s-year-old woman with a history of domestic violence, multiple CT scans of the head following assaults, and death by suicide (from Province of Manitoba archival cohort) (left, original magnification 40x; right, original magnification 200x).


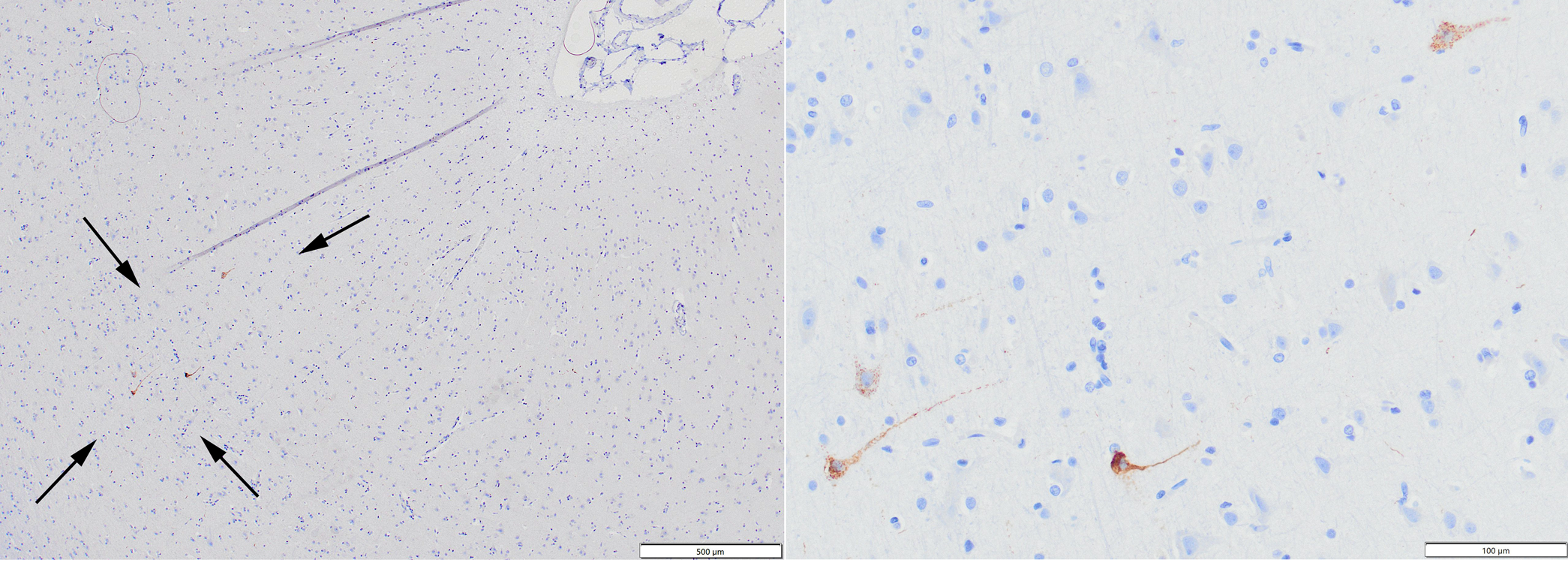


Details of Published Reports of CTE in females

One case report pre-dated the use of p-tau immunohistochemistry; a 76-year-old repeatedly battered woman had brain abnormalities that “resembled that seen in dementia pugilistica” [20]. More recently, perivascular p-tau deposits suggestive of CTE were present in the brain of a 26-year-old woman who had suffered one concussion during school sports and was later repeatedly assaulted by her partner [7]. In two analyses of community autopsy service brains, one showed microscopic CTE-like changes in 3/23 females with histories of head injuries and substance abuse [19] and another showed “features of CTE” in 1 /273 females [2]. Unexpected CTE has generally not been detected in female brains collected in neurodegenerative or suicide brain banks, while ARTAG is prevalent [4, 9, 12, 16, 25]. In one neurodegenerative disease brain bank 32 / 268 cases had early (stage 1 or 2) CTE pathology mainly in the frontal cortex; 13 of the cases were females (age 62-92 years) [14]. All 13 had a history of TBI, and 2/13 (age 68 and 81 years) had histories of domestic assaults (personal communication from author, Dr. Helen Ling, 18 September 2020).

**REFERENCES**

1. Alosco ML, Cherry JD, Huber BR, Tripodis Y, Baucom Z, Kowall NW, Saltiel N, Goldstein LE, Katz DI, Dwyer B et al (2020) Characterizing tau deposition in chronic traumatic encephalopathy (CTE): utility of the McKee CTE staging scheme. Acta Neuropathol 140:495-512. https://doi.org/10.1007/s00401-020-02197-9
2. Bieniek KF, Blessing MM, Heckman MG, Diehl NN, Serie AM, Paolini MA, 2nd, Boeve BF, Savica R, Reichard RR, Dickson DW (2020) Association between contact sports participation and chronic traumatic encephalopathy: a retrospective cohort study. Brain pathology (Zurich, Switzerland) 30:63-74. https://doi.org/10.1111/bpa.12757
3. Bieniek KF, Cairns NJ, Crary JF, Dickson DW, Folkerth RD, Keene CD, Litvan I, Perl DP, Stein TD, Vonsattel JP et al (2021) The second NINDS/NIBIB consensus meeting to define neuropathological criteria for the diagnosis of chronic traumatic encephalopathy. Journal of neuropathology and experimental neurology 80:210-219. https://doi.org/10.1093/jnen/nlab001
4. Bieniek KF, Ross OA, Cormier KA, Walton RL, Soto-Ortolaza A, Johnston AE, DeSaro P, Boylan KB, Graff-Radford NR, Wszolek ZK et al (2015) Chronic traumatic encephalopathy pathology in a neurodegenerative disorders brain bank. Acta neuropathologica 130:877-889. https://doi.org/10.1007/s00401-015-1502-4
5. Braak H, Del Tredici K (2014) Are cases with tau pathology occurring in the absence of Abeta deposits part of the AD-related pathological process? Acta neuropathologica 128:767-772. https://doi.org/ 10.1007/s00401-014-1356-1
6. Crary JF, Trojanowski JQ, Schneider JA, Abisambra JF, Abner EL, Alafuzoff I, Arnold SE, Attems J, Beach TG, Bigio EH et al (2014) Primary age-related tauopathy (PART): a common pathology associated with human aging. Acta neuropathologica 128:755-766. https://doi.org/10.1007/s00401-014-1349-0
7. Danielsen T, Hauch C, Kelly L, White CL (2021) Chronic traumatic encephalopathy (CTE)-type neuropathology in a young victim of domestic abuse. J Neuropathol Exp Neurol 80:624-627. https://doi.org/1093/jnen/nlab015
8. Dawson M (2017) Domestic Homicides and Death Reviews - An International Perspective. Palgrave Macmillan, City, pp 408
9. Forrest SL, Kril JJ, Wagner S, Hönigschnabl S, Reiner A, Fischer P, Kovacs GG (2019) Chronic Traumatic Encephalopathy (CTE) Is Absent From a European Community-Based Aging Cohort While Cortical Aging-Related Tau Astrogliopathy (ARTAG) Is Highly Prevalent. J Neuropathol Exp Neurol 78:398-405. https://doi.org/10.1093/jnen/nlz017
10. Iverson GL, Gardner AJ, Shultz SR, Solomon GS, McCrory P, Zafonte R, Perry G, Hazrati LN, Keene CD, Castellani RJ (2019) Chronic traumatic encephalopathy neuropathology might not be inexorably progressive or unique to repetitive neurotrauma. Brain 142:3672-3693. https://doi.org/10.1093/brain/awz286
11. Iverson GL, Luoto TM, Castellani RJ (2020) Authors' Reply: Age-Related Tau Aggregates Resemble Chronic Traumatic Encephalopathy Neuropathologic Change. J Neuropathol Exp Neurol 79:924-928. https://doi.org/10.1093/jnen/nlaa047.001
12. Koga S, Dickson DW, Bieniek KF (2016) Chronic Traumatic Encephalopathy Pathology in Multiple System Atrophy. J Neuropathol Exp Neurol 75:963-970. https://doi.org/10.1093/jnen/nlw073
13. Kovacs GG, Ferrer I, Grinberg LT, Alafuzoff I, Attems J, Budka H, Cairns NJ, Crary JF, Duyckaerts C, Ghetti B et al (2016) Aging-related tau astrogliopathy (ARTAG): harmonized evaluation strategy. Acta Neuropathol 131:87-102. https://doi.org/10.1007/s00401-015-1509-x
14. Ling H, Holton JL, Shaw K, Davey K, Lashley T, Revesz T (2015) Histological evidence of chronic traumatic encephalopathy in a large series of neurodegenerative diseases. Acta neuropathologica 130:891-893. https://doi.org/10.1007/s00401-015-1496-y
15. Lorigan J, Kearney H, Grimes B, Heffernan J, Beausang A, Cryan J, Farrell MA, Brett FM (2019) Evaluation of the specificity of the central diagnostic criterion for chronic traumatic encephalopathy. Ir J Med Sci 188:993-998. https://doi.org/10.1007/s11845-018-1943-6
16. Matschke J, Sehner S, Gallinat J, Siegers J, Murroni M, Püschel K, Glatzel M (2018) No difference in the prevalence of Alzheimer-type neurodegenerative changes in the brains of suicides when compared with controls: an explorative neuropathologic study. Eur Arch Psychiatry Clin Neurosci 268:509-517. https://doi.org/10.1007/s00406-018-0876-4
17. McKee AC, Stein TD, Crary JF, Bieniek KF, Cantu RC, Kovacs GG (2020) Practical Considerations in the Diagnosis of Mild Chronic Traumatic Encephalopathy and Distinction From Age-Related Tau Astrogliopathy. J Neuropathol Exp Neurol 79: 921-924. https://doi.org/10.1093/jnen/nlaa047
18. Montine TJ, Phelps CH, Beach TG, Bigio EH, Cairns NJ, Dickson DW, Duyckaerts C, Frosch MP, Masliah E, Mirra SS et al (2012) National Institute on Aging-Alzheimer's Association guidelines for the neuropathologic assessment of Alzheimer's disease: a practical approach. Acta Neuropathol 123:1-11. https://doi.org/10.1007/s00401-011-0910-3
19. Noy S, Krawitz S, Del Bigio MR (2016) Chronic Traumatic Encephalopathy-Like Abnormalities in a Routine Neuropathology Service. J Neuropathol Exp Neurol 75:1145-1154. https://doi.org/10.1093/jnen/nlw092
20. Roberts GW, Whitwell HL, Acland PR, Bruton CJ (1990) Dementia in a punch-drunk wife. Lancet 335: 918-919.
21. Schwab N, Hazrati LN (2018) Assessing the Limitations and Biases in the Current Understanding of Chronic Traumatic Encephalopathy. J Alzheimers Dis 64:1067-1076. https://doi.org/10.3233/jad-180373
22. Sinha M (2013) Family violence in Canada: A statistical profile, 2011. Juristat: Canadian Centre for Justice Statistics
23. Smith DH, Dollé JP, Ameen-Ali KE, Bretzin A, Cortes E, Crary JF, Dams-O'Connor K, Diaz-Arrastia R, Edlow BL, Folkerth R et al (2021) COllaborative Neuropathology NEtwork Characterizing ouTcomes of TBI (CONNECT-TBI). Acta Neuropathol Commun 9:32. https://doi.org/10.1186/s40478-021-01122-9
24. Stewart W, Allinson K, Al-Sarraj S, Bachmeier C, Barlow K, Belli A, Burns MP, Carson A, Crawford F, Dams-O'Connor K et al (2019) Primum non nocere: a call for balance when reporting on CTE. The Lancet Neurology 18:231-233. https://doi.org/10.1016/S1474-4422(19)30020-1
25. Walt GS, Burris HM, Brady CB, Spencer KR, Alvarez VE, Huber BR, Guilderson L, Abdul Rauf N, Collins D, Singh T et al (2018) Chronic Traumatic Encephalopathy Within an Amyotrophic Lateral Sclerosis Brain Bank Cohort. J Neuropathol Exp Neurol 77:1091-1100. https://doi.org/10.1093/jnen/nly092
